# Draft Genome Sequences of Nine Environmental Bacterial Isolates Colonizing Plastic

**DOI:** 10.1128/MRA.01485-20

**Published:** 2021-03-18

**Authors:** Ingrid Borre, Eva C. Sonnenschein

**Affiliations:** a Technical University of Denmark, Department of Biotechnology and Biomedicine, Kongens Lyngby, Denmark; Indiana University, Bloomington

## Abstract

Here, we report the draft genome sequences of nine bacterial isolates obtained after laboratory incubation of seawater, soil, and wastewater samples with polylactic acid, polyethylene, or polyethylene terephthalate film for 2 weeks. Assuming colonization as a prerequisite of degradation, these strains could contribute to a solution to the global plastic waste problem.

## ANNOUNCEMENT

Plastic is omnipresent in our environment, and there is no sustainable recycling solution. Many pollutants can be degraded by microorganisms, and degradation is often induced by colonization of the material by the organism. Aiming to contribute a biological solution to the plastic problem, we isolated nine bacterial strains colonizing plastic. Environmental samples (wastewater [WW], seawater [SW], and soil [S]) were obtained from a wastewater treatment plant (Lyngby-Taarbæk Forsyning A/S, Kongens Lyngby, Denmark [55°48′07.4″N, 12°32′20.8″E]) on 14 September 2018, from a harbor (Hellerup, Denmark [55°43′55.5″N, 12°34′51.0″E]) on 17 September 2018, and from a site near the university campus (55°47′07.8″N, 12°30′49.7″E) on 11 October 2018. On the marine and terrestrial sides, plastic pieces (seawater plastic [SWP] and soil plastic [SP]) were also collected. Microbial communities were removed from plastic by sonication for 5 min, and all environmental samples were inoculated at a starting concentration of 10^6^ cells/ml (as determined by SYBR gold staining, filtration, and microscopy) in OECD301 medium (WW, S, and SP samples) or OECD306 medium (SW and SWP samples) (1, 2) with a piece of plastic (18 by 18 mm by 0.05 mm), i.e., poly-l-lactic acid (ME331050; Goodfellow), polyethylene terephthalate (ES301250; Goodfellow), or low-density polyethylene (ET311150; Goodfellow), or a cover glass (18 by 18 mm) (631-1567; VWR) as a control. OECD301 and OECD306 media contain 8.5 mg/liter KH_2_PO_4_, 217.5 mg/liter K_2_HPO_4_, 334 mg/liter Na_2_HPO_4_·2H_2_O, 5 mg/liter NH_4_Cl, 36.4 mg/liter CaCl_2_·2H_2_O, 22.5 mg/liter MgSO_4_·7H_2_O, and 0.25 mg/liter FeCl_3_·6H_2_O in either distilled water (OECD301) or seawater (OECD306) prepared with 2.5% sea salts (Sigma). Cultures were incubated in the dark at 16°C without shaking. After 14 days, the plastic pieces were transferred to fresh OECD301 or OECD306 medium and sonicated for 5 min. The samples were diluted 10-fold and plated onto OECD301 or OECD306 medium with 1.5% agar, 1% polypeptone, and 0.2% yeast extract. After incubation at 16°C for 7 days, colonies that were unique to each plastic type and different from the control samples were isolated by restreaking onto OECD301 or OECD306 1.5% agar plates with 1% polypeptone and 0.2% yeast and incubation for 4 days at 16°C.

Genomic DNA was extracted using the NucleoSpin tissue kit (740952; Macherey-Nagel). Five hundred nanograms of DNA per strain was submitted for sequencing at the Novo Nordisk Center for Biosustainability (Technical University of Denmark, Lyngby, Denmark) using the NextSeq 500/550 midoutput kit v2 (Illumina) for 150-bp paired-end sequencing on a MiSeq Illumina platform. The sequence data were analyzed with KBase v1.8.9 (3). The read quality was assessed using FastQC v0.11.5 (4) and Trimmomatic v0.36 (5) (sliding window size:4; sliding window minimum quality:15; post tail crop length:140; head crop length:10; leading minimum quality: 3; trailing minimum quality:3; minimum read length:36). The genomes were assembled using SPAdes v3.13.0 (6), and quality and metrics were analyzed using QUAST v4.4 (7). The level of contamination was assessed using CheckM v1.0.8 (8). The assemblies were automatically annotated with NCBI PGAP v5.0 (9). Species phylogeny was analyzed using autoMLST (10) (Table 1). Six strains had <95% estimated average nucleotide identity (ANI) with respect to genomes of type strains (11) and thus could represent novel bacterial species.

**TABLE 1:** Genomic features of nine environmental isolates colonizing plastic

Strain	Source	Plastic^*a*^	Closest strain	Estimated ANI (%) to closest type strain	Genome size (bp)	G+C content (%)	Coverage (×)	No. of contigs	*N*_50_ (bp)	No. of genes	SRA accession no.	GenBank accession no.	Assembly accession no.
IB03	WW	PLA	Acidovorax radicis N35^T^	84.6	4,318,166	63.22	243	103	94,511	4,052	SRR13320098	JAEFCH000000000	GCA_016406015.1
IB04	WW	PLA	Pseudomonas veronii DSM 11331^T^	98.1	7,009,445	60.79	164	109	221,072	6,488	SRR13320097	JAEFCG000000000	GCA_016406005.1
IB05	WW	PLA	Paracoccus versutus DSM 582^T^	77.2	5,294,826	63.23	220	108	95,395	5,052	SRR13320096	JAEFCF000000000	GCA_016405985.1
IB15	SW	PLA	Vibrio gigantis LGP 13^T^	90.3	5,060,694	44.22	247	171	67,607	4,659	SRR13320095	JAEFCE000000000	GCA_016406055.1
IB21	SW	PE	*Alteromonas australica* H 17^T^	80.3	4,538,050	44.43	318	78	134,642	3,961	SRR13320094	JAEFCD000000000	GCA_016405965.1
IB30	SWP	PE	Paraglaciecola chathamensis S18K6^T^	97.8	5,067,268	44.18	240	118	125,622	4,389	SRR13320093	JAEILT000000000	GCA_016405925.1
IB36	S	PET	Delftia acidovorans NBRC 14950^T^	98.3	6,368,012	66.86	209	47	292,370	5,770	SRR13320092	JAEFCC000000000	GCA_016405945.1
IB41	S	PE	Variovorax boronicumulans NBRC 103145^T^	89.7	6,867,556	67.50	135	49	283,377	6,448	SRR13320091	JAEFCB000000000	GCA_016405905.1
IB48	SP	PE	Flavobacterium anhuiense CGMCC 16859^T^	89.0	5,603,394	33.47	262	36	663,986	4,730	SRR13320090	JAEFCA000000000	GCA_016405855.1

a PLA, polylactic acid; PE, polyethylene; PET, polyethylene terephthalate.

### Data availability.

The genome assemblies have been deposited in GenBank under BioProject number PRJNA666993, and detailed information is listed in Table 1.
